# Income Tax on "Profits" of Charities

**Published:** 1920-11-13

**Authors:** Chas. H. Tolley


*To the Editor of* The Hospital.

### Income Tax on "Profits" of Charities.

*To the Editor of* The Hospital.

Sir,—It is not commonly appreciated that, strictly speaking, many methods adopted for raising money for charitable and religious purposes (such as periodic sales, entertainments, etc.) are liable to income tax, although it is not usually attempted to assess them.

Quite recently the Rotunda Hospital at Dublin was held to be chargeable with tax on the "profits" of letting rooms for entertainments held in a hall attached to it, and there are a number of other cases. As the law stands, the charitable destination of such profits does not exempt them from tax.

The Government have in preparation a Bill adopting many of the recommendations of the Royal Commission on Income Tax, and it will be noted that the Commissioners do *not* propose exemption of such profits except in the case of "a trade carried on mainly by and for the benefit of the inmates or beneficiaries of a charity." On the contrary, they refuse any concession to "profits earned from undertakings which compete to some extent with ordinary traders."

This seems most unsatisfactory, as it is probable that when this Bill has been introduced and passed the margin of discretion at present allowed to local inspectors will be greatly reduced and the new law strictly followed.

The action of the Revenue with regard to Easter offerings shows that this is no imaginary danger, and all those interested should lose no time in taking action to ensure that the new Bill exempts all purely charitable profits.—Yours faithfully,

Chas. H. Tolley.

